# Ultrasound evaluation of subcentimeter suspicious breast lesions: Correlation with histopathological findings and hormone receptor status

**DOI:** 10.12669/pjms.41.11.11477

**Published:** 2025-11

**Authors:** Nasreen Naz, Ayesha Walid, Anila Rahim, Hira Fatima Waseem

**Affiliations:** 1 Nasreen Naz Professor, Director and Head of Department, Dow Institute of Radiology, Dow University of Health Sciences, Karachi, Pakistan; 2 Ayesha Walid Assistant Professor, Dow Institute of Radiology, Dow University of Health Sciences, Karachi, Pakistan; 3 Anila Rahim Assistant Professor, Dow Institute of Radiology, Dow University of Health Sciences, Karachi, Pakistan; 4 Hira Fatima Waseem Lecturer of Biostatistics, School of Public Health, Dow University of Health Sciences, Karachi, Pakistan

**Keywords:** Hormone Receptor, Solid lesion, Sub-centimeter, Ultrasound

## Abstract

**Background & Objectives:**

Mammography is the routine screening modality for breast cancer detection, however has limitations in dense breast parenchyma and with small lesion size. In these cases, Ultrasound has the potential for early disease detection with lesion size even small than a centimeter, improving patient outcome Primary aim is to evaluate potential of ultrasound imaging in characterizing suspicious sub-centimeter solid breast lesions by using BIRADS lexicon descriptors and correlate with histopathology. Secondary aim is to evaluate differences in sonographic appearance of different molecular subtypes of invasive sub centimeter malignancy.

**Methods:**

In this retrospective study sub-centimeter solid lesions diagnosed as BIRADS IV-V, between 2020 to 2023 were included at Dow Institute of Radiology, Karachi, Pakistan. Lesion shape, margins, posterior features, orientation, vascularity and calcifications were reviewed from database. Univariate and multivariable binary logistic regression analysis performed to determine significant malignant findings on ultrasonography. Morphological features specific to molecular subtypes was assessed. A p-value of ≤ 0.05 was used to report statistical significance.Differences in sonographic appearance of molecular subtypes of invasive malignancy were seen in 90 sub-centimeter lesions.

**Results:**

Out of 90 lesions, 51 were malignant, and 39 were benign. On multivariable analyses, irregular shape, hypoechoic echotexture and not parallel orientation were significant features to differentiate between benign and malignant disease. No meaningful difference in sonographic appearance of molecular subtypes was observed in sub centimeter lesions

**Conclusion:**

Ultrasound is a beneficial modality for detection of sub-centimeter breast lesions, allowing appropriate BIRADS categorization. With decreasing lesion size, morphological characterization becomes difficult and needs careful evaluation. Irregular shape and not parallel orientation were the predictive sonographic signs of malignancy in sub-centimeter lesions.

## INTRODUCTION

Breast cancer remains the most prevalent health issue affecting women worldwide. In Pakistan, incidence of breast cancer is showing an alarming rise, with over 90,000 cases annually and one in nine women at risk.^1^,^2^ While mammography aids in early detection, it struggles with dense breast tissue and small tumors. Research shows ultrasound improves detection of mammographically hidden masses by 27%, particularly in women under 50 years highlighting its value in enhancing breast cancer screening and diagnosis.^3^ Studies by Berg WA et al. have shown that cancers found only by ultrasound were invasive, small (median size of 12 mm), and node negative (64.2%).^4^

Single center studies have reported that 28% of breast cancer patients in Pakistan at the time of diagnosis are below age of 40, when mammographic screening is not performed.^5^,^6^ In these cases, ultrasound is the primary imaging method for detecting disease and accurately characterizing lesions. Breast cancer treatment depends on tumor size and nodal status with increase in awareness and imaging advancements, women are being diagnosed with tumors even less than 1 cm in size, when the disease is usually non palpable.^7^ It is need of time to accurately characterize these subcentimeter lesions to improve the decision-making process and patient outcome.

Correlating ultrasound features of breast cancer with hormone receptor status helps predict tumor biology, and support personalized patient care. This can guide treatment planning especially in developing countries with limited laboratory and financial resources. International and local data is evolving in this respect is mostly limited to large size lesions. Local study by Shaikh et al.^8^ has shown that triple negative malignancy commonly shows posterior enhancement. Another recent international study with similar results also shows that luminal subtypes are commonly spiculated vascular lesions with posterior shadowing.^9^ We aimed to see for any meaningful difference in sonographic appearance of sub-centimeter malignancy. These results can also guide towards development of radiomics and AI tools.

Limited research describes the imaging features of Sub-centimeter breast lesions on different modalities with no published data from our country. Our study aimed to analyze ultrasound features of Sub-centimeter solid breast lesions, distinguish benign from malignant, and evaluate hormonal receptor status as potential diagnostic markers for Sub-centimeter malignancy.

## METHODOLOGY

This retrospective descriptive, observational study was conducted at Dow Institute of Radiology,. Electronic ultrasound reports between July 2020 to August 2023 were reviewed from HMIS. Patients diagnosed with Sub-centimeter solid lesions on ultrasound and categorized as either BIRADS four or five, having subsequent ultrasound guided biopsy and histopathology at our hospital were recruited as study population. Male patients, cystic lesions, breast implants, known multicentric / multifocal breast malignancy and incomplete data were excluded.

### Ethical Approval:

It was received from IRB Ref. IRB 3165/DUHS/EXEMPTION/2023/336; Dated: September 4, 2023 Informed consent was waived.

### Imaging and interpretation:

Breast ultrasounds were performed with 5-14 MHz linear probe (Toshiba APLIO 300) and core-needle biopsies acquired using a 16-gauge needle by a breast radiologist with minimum seven years of experience. Ultrasound images were acquired following a standard protocol and stored on Picture Archiving and Communication System (PACS) for every patient. Lesions were classified using Breast Imaging Reporting and Data System (BIRADS), updated in 2014 by the American college of Radiology (ACR). According to category five assessment imaging features represents ≥95% likelihood of malignancy. Category four represents a wide range of 2-94%, further classified as 4a (2-10%, low risk), 4b (10-50%, moderate risk) and 4c (50-94%, tyhigh risk).^8^,^9^ Patient demographics, clinical presentation and side of lesion were recorded from reports on HMIS. Ultrasound images from PACS reviewed to record morphological lesion descriptors as, shape, margins, posterior features, orientation, vascularity and intralesional calcifications. Lesion size was measured as the maximum dimension in mm. Axillary lymph nodes with cortical thickening greater than 3 mm were regarded suspicious.^9^

### Histopathology and immunohistochemistry analysis:

Laboratory data was also archived from the database (HMIS) and taken as reference standard. Histopathology categorized malignant lesions as ductal carcinoma in-situ (DCIS), invasive ductal carcinoma (IDC), invasive lobular carcinoma (ILC), and invasive papillary carcinoma. Grade was defined according to modified Scarff-Bloom-Richardson system (7) as: grade-1 (well-differentiated), Grade-2 (moderately differentiated) and Grade-3 (poorly differentiated). Grades-1 and 2 were considered as low grade and Grade-3 as high grade in this study.

Immunohistochemistry (IHC) evaluated expression of estrogen receptor (ER), progesterone receptor (PR), human epidermal growth factor two (HER2) on the cell surface, and Ki-67 proliferation index. Molecular subtypes were defined in accordance to the St. Gallen International Expert Consensus guidelines as: Luminal A (LA): ER+, PR+, HER2-, and low Ki-67 index; luminal B (LB): ER+, PR+ or PR-, HER2-, and high Ki-67 index; HER2- enriched: ER-, PR-, HER2+; and triple-negative (TN): ER-, PR-, HER2-.^10^

### Statistical Analysis:

Data analysis utilized SPSS version 26.0. Categorical variables were summarized with frequencies/percentages, and medians with interquartile ranges for non-normal continuous data. Univariate analysis (Pearson χ², Fisher’s exact test) and binary logistic regression were performed to estimate the effects of covariates on benign and malignant outcomes, reporting crude odds ratios (OR) with 95% confidence intervals. Covariates with p < 0.20 were included in multivariable analysis, adjusted odds ratios (OR) with 95% confidence intervals were reported (Table-I). BI-RADS positive predictive value (PPV) was calculated for cancer detection rates (number of breast cancers divided by total number of examinations per category × 100) (Table-II). Furthermore, Pearson χ² and Fisher’s exact test were performed for demographics and ultrasound characteristics according to IHC classification of malignant lesions (Table-III). A value of p < 0.05 was considered as significant.

**Table-I:** Patients and their ultrasound characteristics of Benign and Malignant Lesions.

Characteristics		Benign n=39 (%)	Malignant n=51 (%)	Total n=90(%)	p-value	Crude OR (95% CI)	p-value	Adjusted OR (95% CI)	p-value
***Age (years)***									
	≤47 years	25(64.1)	23(45.1)	48(53.3)	0.073	1		-	-
	>47 years	14(35.9)	28(54.9)	42(46.7)		2.17 (0.92-5.11)	0.075		
***Tumor Size (mm)***									
	≤7 mm	24(61.5)	28(54.9)	52(57.8)	0.528	1		-	-
	>7 mm	15(38.5)	23(45.1)	38(42.2)		1.31 (0.56-3.07)	0.528		
***Side***									
	Left	21(53.8)	22(43.1)	43(47.8)	0.314	1		-	-
	Right	18(46.2)	29(56.9)	47(52.2)		1.53 (0.66-3.55)	0.315		
***Location***									
	Upper outer/inner	24(61.5)	33(64.7)	57(63.3)	0.722	1		-	-
	Lower outer/inner	9(23.1)	13(25.5)	22(24.4)		1.05 (0.38-2.85)	0.923		
	Retroareolar	6(15.4)	5(9.8)	11(12.3)		0.60 (0.16-2.22)	0.450		
***Family history***									
	Negative	29(74.4)	37(72.5)	66(73.3	0.847	1		-	-
	Positive	10(25.6)	14(27.5)	24(26.7)		1.09 (0.42-2.82)	0.847		
***Presentation***									
	Without complaint	9 (23.1)	7 (13.7)	16 (17.8)	0.250	1		-	-
	With complaint	30 (76.9)	44 (86.3)	74 (82.2)		1.88 (0.63-5.61)	0.255		
		***Ultrasound characteristics***							
***Shape***									
	Irregular	12 (30.8)	48 (94.1)	30 (33.3)	<0.001*	1		1	
	Oval/round	27 (69.2)	3 (5.9)	60 (66.7)		0.02 (0.01-0.10)	<0.001*	0.03 (0.01-0.28)	0.002
***Margins***									
	Non circumscribed	26(66.7)	50(98.0)	76(84.4)	<0.001*	1		1	
	Circumscribed	13(33.3)	1(2.0)	14(15.6)		0.04 (0.01-0.32)	0.003*	0.79 (0.04-14.01)	0.876
***Posterior features***									
	Enhancement	31(79.5)	39(76.5)	70(77.8)	0.733	1			
	Shadowing	8(20.5)	12(23.5)	20(22.2)		1.19 (0.43-3.27)	0.733	-	-
***Orientation***									
	Not parallel	5(12.8)	34(66.7)	39(43.3)	<0.001*	1		1	
	parallel	34(87.2)	17(33.3)	51(56.7)		0.07 (0.02-0.22)	<0.001*	0.14 (0.02-0.74)	0.021
***Echotexture***									
	Hypoechoic	19(48.7)	38(74.6)	57(63.3)	0.004*	1		1	
	Hyperechoic	14(35.9)	4(7.8)	18(20.0)		5.25 (1.15-23.93)	0.032*	13.90 (1.48-129.75)	0.021
	Heterogenous	6(15.4)	9(17.6)	15(16.7)		7.00 (2.02-24.19)	0.002*	1.65 (0.22-12.29)	0.622
***Vascularity***									
	Absent	16(41.0)	18(35.3)	34(37.8)	0.008*	1		1	
	Predominant peripheral	9(23.1)	2(3.9)	11(12.2)		0.19 (0.03-1.05)	0.058*	0.60 (0.03-10.27)	0.725
	Predominant central	14(35.9)	31(60.8)	45(50.0)		1.98 (0.78-4.95)	0.150	3.34 (0.75-14.88)	0.113
***Intralesional calcifications***									
	Absent	28(71.8)	43(84.3)	71(78.9)	0.149	1			
	Present	11(28.2)	8(15.7)	19(21.1)		0.47 (0.16-1.32)	0.154	0.23 (0.05-1.09)	0.064

**Table-II:** PPV of Ultrasound BIRADS category.

	Benign	Malignant	Total	PPV (%)
4a	30	2	32	6.25
4b	9	9	18	50.0
4c	0	25	25	100.0
5	0	15	15	100.0
Total	39	51	90	

**Table-III:** Demographics and ultrasound characteristics according to IHC classification of malignant lesions.

		IHC classification of malignant lesions (n=51)					p-value
Characteristics		DCIS (n=6)	Luminal A (n=17)	Luminal B (n=12)	HER 2 enriched (n=4)	Triple Negative (n=12)	
		n (%)	n (%)	n (%)	n (%)	n (%)	
***Age (years)***							
	≤47 years	3(50.0)	6(35.3)	6(50.0)	1(25.0)	7(58.3)	0.687
	>47 years	3(50.0)	11(64.7)	6(50.0)	3(75.0)	5(41.7)	
***Tumor Size (mm)***							
	≤7 mm	5(83.3)	11(64.7)	7(58.3)	2(50.0)	3(25.0)	0.139
	>7 mm	1(16.7)	6(35.3)	5(41.7)	2(50.0)	9(75.0)	
***Axillary nodal metastasis***							
	Absent	5(83.3)	13(76.5)	10(83.3)	4(100.0)	6(50.0)	0.279
	Present	1(16.7)	4(23.5)	2(16.7)	0(0.0)	6(50.0)	
***Histopathology***							
	Invasive ductal	-	14(82.4)	11(91.7)	4(100.0)	12(100.0)	NA
	Invasive lobular	-	2(11.8)	1(8.3)	0(0.0)	0(0.0)	
	Invasive papillary	-	1(5.9)	0(0.0)	0(0.0)	0(0.0)	
***Grade***							
	Low	4(66.7)	15(88.2)	11(91.7)	1(25.0)	3(25.0)	<0.001*
	High	2(33.3)	2(11.8)	1(8.3)	3(75.0)	9(75.0)	
***Shape***							
	Irregular	5(83.3)	17(100.0)	11(91.7)	4(100.0)	11(91.7)	NA
	round/oval	1(16.7)	0(0.0)	1(8.3)	0(0.0)	1(8.3)	
***Margins***							
	Non-circumscribed	5(83.3)	17(100.0)	12(100.0)	4(100.0)	12(100.0)	NA
	Circumscribed	1(16.7)	0(0.0)	0(0.0)	0(0.0)	0(0.0)	
***Posterior features***							
	Enhancement	6(100.0)	10(58.8)	9(75.0)	3(75.0)	11(91.7)	0.176
	Shadowing	0(0.0)	7(41.2)	3(25.0)	1(25.0)	1(8.3)	
***Orientation***							
	Not parallel	0(0.0)	13(76.5)	10(83.3)	2(50.0)	9(75.0)	0.003*
	Parallel	6(100.0)	4(23.5)	2(16.7)	2(50.0)	3(25.0)	
***Echotexture***							
	Hypoechoic	1(16.7)	15(88.2)	10(83.3)	3(75.0)	9(75.0)	NA
	Hyperechoic	2(33.3)	1(5.9)	1(8.3)	0(0.0)	0(0.0)	
	Heterogeneous	3(50.0)	1(5.9)	1(8.3)	1(25.0)	3(25.0)	
***Vascularity***							
	Absent	2(33.3)	4(23.5)	5(41.7)	3(75.0)	4(33.3)	NA
	Peripheral	1(16.7)	0(0.0)	1(8.3)	0(0.0)	0(0.0)	
	Central	3(50.0)	13(76.5)	6(50.0)	1(25.0)	8(66.7)	
***Intralesional calcifications***							
	Absent	3(50.0)	14(82.4)	10(83.3)	4(100.0)	12(100.0)	NA
	Present	3(50.0)	3(17.6)	2(16.7)	0(0.0)	0(0.0)	

p-value calculated using χ2 and Fisher’s exact test.

* p < 0.05.

## RESULTS

This study analyzed 90 sub-centimeter breast lesions, with a median patient age of 47 years (IQR: 38-55) and lesion size of 7 mm (IQR: 6-8). Histopathology identified 43.3% benign and 56.7% malignant lesions. No significant association was observed between patient characteristics (age, tumor size, side, location, family history, presentation) and malignancy (p > 0.05). Significant sonographic predictors of malignancy included irregular shape, non-circumscribed margins, hypoechoic echotexture, and central vascularity (p < 0.05), while parallel orientation correlated with benignity (p < 0.001). Posterior enhancement (77.8%) and absence of calcifications (78.9%) showed no meaningful difference between benign and malignant groups. Significant sonographic features were additionally subjected to multivariate logistic regression analysis.

Irregular shape, hypoechoic echotexture and not parallel orientation were significant variables in the malignant group. The patients’ characteristics and lesion sonographic descriptors according to benign/malignant group are detailed in Table-I. Most common benign lesion was intra ductal papilloma (28.9%) on histopathology (Graph-1). Our study yielded PPV of 6.25%, 50% 100% and 100% for BIRADS category 4a, 4b, 4c and 5 respectively, when correlated with histopathology (Table-II). Only grade and orientation significantly associated with IHC classification of malignant lesions (p<0.05). Patients’ characteristics and lesion descriptors according to IHC classification of malignant lesions are detailed in Table-III.

Molecular subtype was not mentioned in pathology reports of patients with DCIS, hence it was taken as a distinct entity. IDC accounted for 80.4% of malignancies and LA was the most common molecular subtype (33.3%) DCIS lesions were smaller (5.60 ± 1.67 mm) and significantly exhibited parallel orientation (*p=.003*)). A high grade was significantly observed in TN and Her 2 enriched subtypes (75%) while DCIS and Luminal subgroups were frequently low-grade lesions. Unfortunately, there was no statistically significant difference between the subtypes with respect to other ultrasound features in our study. However, it was observed that LA and LB subtypes presented more often as hypoechoic lesions (88.2% and 83.3% respectively) with central vascularity (Fig.2), Axillary nodal metastasis showed high rates in TN subgroup (50%). DCIS showed high percentage of intralesional calcifications (50%) appearing commonly heterogeneous (50%) with increased central vascularity (60%) and posterior enhancement (100%). Spiculated margins seen most frequently in all subtypes of invasive cancer were not seen in any case of DCIS, rather 66.7% of DCIS presented with angular margins.

## DISCUSSION

As diagnostic methodologies evolve, there is a growing emphasis on detecting even the smallest suspicious breast lesion. Wide availability of US has increased the incidental detection of Sub-centimeter lesions which could be either benign or malignant, hence accurate morphological characterization remains challenging for radiologists. ACR BIRADS-US lexicon provides a standardized categorization system for lesion morphology, with limited variable results in Sub-centimeter lesions.

Sonographic features showing meaningful differentiation between benign/malignant Sub-centimeter lesions in our study were shape, margins, echotexture, orientation and pattern of vascularity, allowing appropriate BIRADS categorization. Irregular shape, hypoechoic echotexture and not parallel orientation were most important sonographic signs of invasive Sub-centimeter malignancy in our study. This finding is comparable with study of Elverici et al.^3^ in which 186 non-palpable BIRADS 4 lesions with mean diameter of 9.8 mm were evaluated and irregular shape was found associated with a 6.56-fold increase in risk of malignancy.

**Fig.1:**
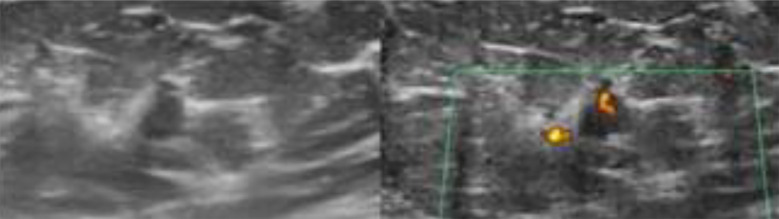
Focused ultrasound image of right breast demonstrating a 8 mm solid vascular lesion with spiculated margins with posterior enhancement. Proved IDC grade-2 on histopathology.

We have observed spiculated margins in 51% of Sub-centimeter malignancy followed by angular margins in 27.4% lesions. (Fig.1). Commonly breast malignancy is anticipated with spiculated margins. Recent study by Fidan et al.^7^ concludes that the most frequent ultrasound sign was spiculated and irregular-indistinct margins (34.9%) in small malignant lesions. At the same time, we observed that non circumscribed margins were seen with increasing frequency in Sub-centimeter lesions with benign histopathology, similar to studies by Elverici et al.^3^ and Fidan et al.^11^, emphasizing that it is difficult to differentiate margin characteristics with decreasing lesion size, interobserver variability and radiologist expertise also playing crucial role in decision making. Posterior shadowing is not significant sonographic feature in this study, possibly secondary to low desmoplastic reaction and less heterogeneous echotexture in Sub-centimeter lesions. However, this hall mark sign of malignancy, is commonly seen in lesions > 2 cm and majority of Her-2 enriched tumors in the study conducted by Shaikh et al.^8^ In the present study, cancer predictive value of BIRADS 4a, 4b, 4c and 5 were 6.25, 50, 100 and 100% respectively. For BI-RADS 4a, 4b and 5, our findings are comparable to the likelihood of malignancy rate as per fifth edition BI-RADS atlas.^10^,^12^ No difference in PPV of 4c and 5 categories in our study noted, probable reason being difficult morphological characterization in Sub-centimeter lesions and other being non rigid ACR criteria for sub classification of category four.

**Fig.2:**
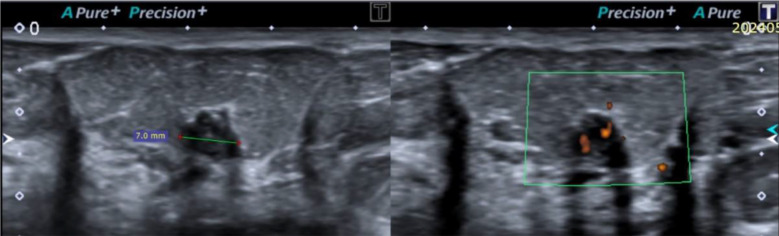
A 7 mm solid hypoechoiec lesion with angular margins, posterior enhancement and central feeding vessel is seen in right breast. Also shows few echogenic foci / calcifications. Biopsy shows IDC grade-3.

**Fig.3:**
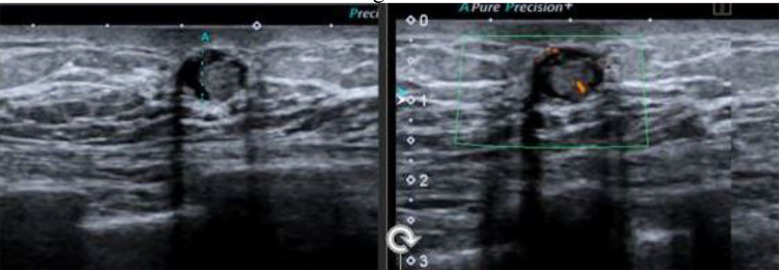
Biopsy-proven IDC grade-2 presents as a 6 mm solid appearing heterogeneous echotexture lesion showing posterior enhancement and central feeding vessel in the left breast.

**Fig.4:**
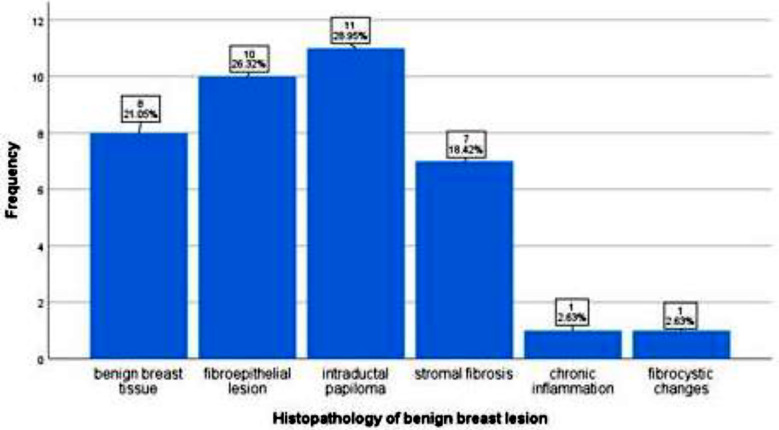
Distribution of benign lesions according to histopathology.

DCIS, an early noninvasive malignancy, presented with overlapping signs of parallel orientation, heterogeneous echotexture, posterior enhancement and increased vascularity in our current study. Yao et al. studied 296 DCIS lesions on ultrasound and concluded that it was difficult to detect ≤ 10 mm lesions on ultrasound as these presented with atypical signs that could lead to missed diagnosis. In their study 22% of DCIS lesions were underestimated based on the ultrasound BI-RADS assessment.^13^

We report a relatively high frequency of TN variety (23.5%) in our population, which is similar to results of Shaikh et al.^8^ TN subtype, known for worst prognosis usually constitutes 10-20% of breast malignancy in prior studies^10^,^14^,^15^ Consistent with literature, IDC was the most common histologic variety (80%) and LA (33.3%) most prevalent molecular subtype.^10^,^15^ Advances in molecular biology have enabled personalized breast cancer treatment, making the identification of subtypes through sonographic features crucial. Limited conducted research focuses on large size lesions. Khalaf et al. and Rashmi et al.^15^,^16^ evaluated lesions with mean size of 3 cm and demonstrated that luminal subtypes present with non-circumscribed margins and posterior shadowing. Microcalcification, posterior mixed acoustic features, and high vascularity are predictive sonographic signs of HER2 subtype. Nodal metastasis is the strongest independent predictor for HER2, and its absence predictive for LA subtype. Studies have proved that low grade tumors result in spicules and well demarcated margins are common with high grade tumors.^13^,^17^,^18^ Our study also supports this observation as luminal subtypes were frequently low grade and Her2 enriched and TN groups were high grade tumors.

### Strength of the study:

This study emphasizes that dedicated radiological expertise can diagnose breast malignancy when Sub-centimeter in size on ultrasound. There is overlap of sonographic descriptors in Sub-centimeter lesions, hence accurate assessment requires analysis of multiple feature descriptors. Detection of small malignant mass is important to allow the patient to benefit from earlier diagnosis and the curable on time treatment, potentially leading to cost savings for healthcare systems. This study provides a basis for future research on elasticity assessment of Sub-centimeter lesions for distinguishing between benign and malignant cases.

### Limitations:

Firstly, ultrasound features of the lesions were recorded from static images due to retrospective study design. Real time ultrasound allows for more detailed information. Secondly, the results cannot be generalized, limited by small sample size and single institution experience.

## CONCLUSION

We conclude that irregular shape, hypoechoic echotexture and not parallel orientation are the most important sonographic signs of invasive sub-centimeter malignancy. It is difficult to detect sonographic differences between molecular subtypes in sub-centimeters lesions. DCIS presents with atypical sonographic appearance, so high radiological suspicion in clinical context will prevent missed diagnosis.

### Authors’ Contributions:

**NN:** Conception and design, final approval of the version to be published.

**AW:** Acquisition of data, drafting the article.

**AR:** Acquisition of data, revising the article, critical approval of intellectual content.

**HFW:** Analysis and interpretation of data, second draft review of the manuscript.

All authors have read and approved the final version of the manuscript. They are also accountable for the integrity of the study.
